# How do nanoparticle properties shape pharmacokinetics and pharmacodynamics? A mechanistic review

**DOI:** 10.3389/fphar.2025.1704814

**Published:** 2026-02-10

**Authors:** Esperanza Peralta-Cuevas, Nahomi Y. Degollado-Hernández, Iliana C. Martínez-Ortiz, Ashley J. Gutierrez-Onofre, Igor Garcia-Atutxa, Francisca Villanueva-Flores

**Affiliations:** 1 Centro de Investigación en Ciencia Aplicada y Tecnología Avanzada (CICATA) Unidad Morelos del Instituto Politécnico Nacional (IPN), Xochitepec, Mexico; 2 Universidad Tecnológica Emiliano Zapata del Estado de Morelos, Emiliano Zapata, Morelos, Mexico; 3 Departamento de Ciencias de la Computación, Universidad Católica de Murcia (UCAM), Murcia, Spain

**Keywords:** nanoparticles, pharmacodynamics, pharmacokinetics, protein corona, transcytosis

## Abstract

Many therapeutics fail due to suboptimal pharmacokinetics/pharmacodynamics (PK/PD), and the development of nanomedicine remains partly empirical. We present a protocol-guided, mechanistic review that links nanoparticle (NP) physicochemical traits (such as size, surface charge/hydrophobicity, elasticity, and composition) to protein-corona remodeling, immune recognition, biodistribution, and clearance across the ADME, and examines how these shifts translate into pharmacodynamics and therapeutic index. We systematically searched PubMed, Web of Science, Scopus, and ClinicalTrials.gov (primarily 2000–2025) using predefined Boolean strings. We included *in vivo* animal or human studies directly comparing the same drug in NP *versus* non-NP formulations with matched route and comparable dose, reporting at least one PK and/or PD endpoint, while excluding in vitro-only reports, reviews/opinion articles, and studies with undisclosed or non-comparable vehicles. We curated *in vivo*, route-matched comparisons of the same drug in NP *versus* non-NP forms and compiled an eight-drug cross-compound dataset. Where variance permitted, we quantified effects using standardized ln-ratios for AUC, half-life, and clearance, and flagged vehicle-matched comparisons to avoid overattributing gains to nanoscale architecture. Across oral and intravenous delivery, NPs consistently increased systemic exposure and prolonged half-life, with the most significant benefits in poorly soluble, lipophilic molecules; effect sizes were context-dependent and, in some formulations, reached multi-fold improvements. Mechanistically, we delineate when lymphatic uptake (long-chain lipid carriers), receptor-mediated transcytosis (e.g., BBB-oriented designs), or context-limited EPR are most contributory, and we highlight stiffness-dependent corona effects (e.g., ApoA-I enrichment) as a lever to extend circulation. To enable translation, we contribute: (i) a mechanism-to-metric map that connects trait tuning to PK readouts and PD/TI implications; (ii) a standardized, route-stratified table of ln-ratio effects with explicit vehicle/excipient flags; and (iii) a pragmatic decision framework to triage candidates (by logP/solubility and barrier goals) and design reproducible studies with safety checks (CARPA, anti-PEG/ABC, inorganic retention). Collectively, these elements advance anticipatory, model-informed nanoformulation design and provide quantitative anchors for future vehicle-matched, mechanism-driven comparisons.

## Graphical Abstract

**Figure:**
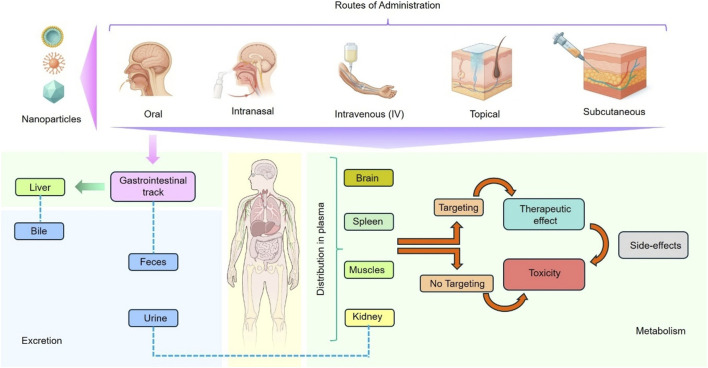

## 1 Introduction

A striking proportion of promising therapeutic molecules fail to reach clinical use due to inadequate pharmacokinetic/pharmacodynamic (PK/PD) profiles (Dowden and Munro, 2019; Harrison, 2016). Drugs that are potent *in vitro* often suffer from poor solubility, rapid clearance, or off-target distribution *in vivo*, resulting in subtherapeutic exposure or unacceptable toxicity. Nanotechnology-based drug delivery has emerged as a potential solution to these issues: nanoparticles can prolong systemic circulation, enhance tissue-specific drug delivery, and improve drug stability and solubility (Gabizon et al., 2003; Suk et al., 2016; Villanueva-Flores et al., 2020). For example, encapsulating doxorubicin in a PEGylated liposomal nanoparticle (Doxil®) dramatically increases its circulation time and tumor accumulation, enabling higher effective doses with significantly reduced cardiotoxicity (Yuan et al., 2019). Such successes highlight the need to understand better how nanocarriers alter drug fate, beyond the trial-and-error strategy that has gained traction in drug development.

Despite their promise, nanoformulations introduce new levels of complexity that demand a shift from empirical to mechanistic insight. Nanoparticles often exhibit distinct and complicated *in vivo* disposition compared to conventional drug forms (Yuan et al., 2019). A myriad of physicochemical parameters, particle size, shape, surface charge, composition, and surface chemistry, to name a few, can profoundly influence how nanoparticles interact with biological systems and distribute among tissues (Yuan et al., 2019; Cao et al., 2020; Tekie et al., 2020). Moreover, nano-carriers are not always inert vehicles; they can actively engage biological pathways. For instance, specific nanomaterials can modulate the pathological environment by reducing inflammation and releasing therapeutic ions or reactive species, as demonstrated in chronic wound models (Pazyar et al., 2014; Díaz-Barriga et al., 2021; Villanueva-Flores et al., 2023a). Traditional PK measures (such as plasma drug levels) often fail to capture these multifaceted behaviors; a drug’s blood concentration alone cannot reveal its nanoparticle-mediated tissue targeting or local bioactivity (Haripriyaa and Suthindhiran, 2023; Villanueva-Flores et al., 2023b; Nájera-Maldonado et al., 2025). Consequently, there is a critical need to move beyond *ad hoc* optimization and toward a mechanistic framework that links nanoparticle design to *in vivo* drug behavior. Recent advances in physiologically based pharmacokinetic modeling underscore this need; mechanistic models are now predicting nanoparticle biodistribution and drug effects by accounting for biological transport processes and nano–bio interactions. Embracing such quantitative, mechanism-driven approaches will enable researchers to anticipate efficacy and toxicity outcomes rather than retrospectively rationalizing them.

In this review, we consolidate and critically appraise mechanistic evidence on how nanoparticle physicochemical properties, carrier architectures, and administration routes influence each stage of the ADME process and shape the pharmacodynamic response. We organize recurring, mechanistically grounded trends, such as size-dependent clearance pathways, charge-modulated protein corona composition, and elasticity-linked biodistribution, and, where primary data permit, report representative quantitative ranges rather than assert universal thresholds or predictive models. Our contribution is a structured mechanism-to-outcome framework, with practical design heuristics and common pitfalls anchored in traceable case studies, to guide formulation, triage, and experimental planning. We also delineate domains where evidence is contradictory or sparse (e.g., magnitude of EPR in humans, *in vivo* corona dynamics, long-term safety trade-offs), thereby bounding expectations and outlining the specific studies needed to advance toward truly predictive nanotherapeutic design.

## 2 Methods

### 2.1 Review design and information sources

We conducted a protocol-guided, mechanistic review that maps nanoparticle (NP) physicochemical traits (size, shape, surface charge/hydrophobicity, elasticity, composition) to nano–bio interactions and consequences for pharmacokinetics (CL, t½, AUC) and pharmacodynamics/therapeutic index. To illustrate quantitative patterns, we assembled an eight-drug cross-compound dataset contrasting NP formulations with matched conventional comparators. Information was retrieved from PubMed/MEDLINE, Web of Science, Scopus, and ClinicalTrials.gov, limited primarily to 2000–2025, with inclusion of earlier sentinel papers when foundational to mechanisms; English and Spanish sources were considered. Representative Boolean strings (adapted per database/field tags) included (“nanoparticle*” OR liposom* OR micell* OR “polymeric nanoparticle*” OR “solid lipid nanoparticle*”) AND (AUC OR “half-life” OR clearance OR bioavailability OR “%ID/g” OR “tumor accumulation”) AND (IV OR oral) (“protein corona” OR apolipoprotein OR “ApoA-I”) AND (stiffness OR elasticity OR modulus) AND (clearance OR “half-life” OR biodistribution); and (nanoparticle* AND (“blood–brain barrier” OR BBB OR transcytosis)) AND (receptor-mediated OR adsorptive). Database-specific syntaxes (e.g., PubMed [tiab]/MeSH; Scopus TITLE-ABS-KEY; Web of Science TS = with proximity operators). The last search was completed on 13 August 2025.

### 2.2 Eligibility, data extraction, and synthesis

We included *in vivo* animal or human studies evaluating the same drug as NP *versus* a relevant non-NP comparator with matched route (intravenous or oral) and comparable dose (±20% or justified), reporting at least one PK endpoint (AUC, t½, CL, or bioavailability) and/or an objective PD outcome; *in vitro* studies, reviews/opinions, and reports with undisclosed vehicles preventing attribution were excluded. Titles/abstracts were screened in duplicate with third-reviewer adjudication; full texts were examined with documented reasons for exclusion. The eight-drug subset was pre-specified to span lipophilicity/solubility space (BCS II/IV) and diverse carrier classes with traceable PK/PD. Two reviewers independently extracted species, model, route, dose, sample size, NP class and characterization (size, PDI, ζ-potential, morphology, coatings/ligands, elasticity when reported), comparator and vehicle/excipients, PK parameters (AUC, t½, CL, Cmax, F), distribution metrics (%ID/g or tissue AUC), and PD outcomes; units were harmonized and figures digitized when necessary with documented assumptions. The primary effect size was the log-ratio of AUC [ln (AUC_NP/AUC_control)], with secondary ln-ratios for t½, CL (sign-inverted), and F (oral); distribution effects were summarized as ln-ratios of %ID/g or tissue AUC, and PD as relative or absolute change in the study’s primary efficacy endpoint. Nanomedicine-specific confounders were explicitly flagged, including vehicle matching (e.g., TPGS/surfactants as active excipients), completeness of NP characterization, route/dose alignment, adequacy of sampling windows for long half-lives, and plausibility checks for outlier measurements. Risk of bias in animal studies was appraised with SYRCLE domains (graded low/high/unclear); human evidence was qualitatively assessed for randomization, blinding, comparator adequacy, attrition, and registration. Given heterogeneity across species, routes, and vehicles, we used semi-quantitative synthesis: individual study ln-ratios are reported, and where at least three studies were comparable (same route/species and vehicle-matched), medians with interquartile ranges are provided without parametric pooling.

### 2.3 Influence of nanoparticle physicochemical properties on pharmacokinetics and pharmacodynamics

Size plays a key role in protein corona formation and clearance: for example, ∼80 nm particles adsorb significantly fewer opsonins than larger 170–240 nm particles, leading to roughly half the clearance rate (i.e., longer circulation) for the 80 nm particles (Villanueva-Flores et al., 2020; Ernsting et al., 2013). Tiny nanoparticles below ∼five to eight nm are rapidly filtered out by the kidneys (Longmire et al., 2008), whereas very large nanoparticles above ∼200 nm tend to be trapped and removed by the spleen’s macrophages (Hirn et al., 2011). Particles around 100 nm often achieve an optimal balance; they avoid immediate renal clearance and spleen sequestration, thus persisting longer in blood and efficiently accumulating in leaky tumor tissues *via* the EPR effect (Ernsting et al., 2013). Shape further modulates these outcomes by altering both protein adsorption and cell uptake. Rod-shaped nanoparticles, for instance, bind a greater total mass of plasma proteins than equal-sized spheres and show a different composition in their corona (enriching immunoglobulins vs. albumin) (Madathiparambil et al., 2020). Non-spherical (rod-, worm-, or discoid-like) carriers often show prolonged circulation and altered coronas relative to equal-volume spheres; effects vary with material, stiffness, and surface chemistry, so we do not assert fixed multipliers (Ernsting et al., 2013). These geometric factors influence where nanoparticles distribute (spheres tend to distribute evenly, whereas rods may migrate to vessel walls or even lodge in specific organs) and how effectively they reach target tissues, ultimately impacting the pharmacodynamic outcome of the delivered therapy.

The surface chemistry of nanoparticles, particularly charge and hydrophobicity, governs protein-corona composition and immune interactions, thereby affecting circulation time and cellular uptake. Charged surfaces recruit oppositely charged blood proteins; for example, cationic nanoparticles readily bind negatively charged proteins and cell membranes, promoting opsonin attachment and immune recognition. Highly cationic or highly anionic particles are often rapidly opsonized and cleared by Kupffer cells. In contrast, particles whose effective surface after corona formation is near-neutral (zeta potential ≈ ±10 mV) and sufficiently hydrophilic/“stealth” (e.g., PEGylated or zwitterionic coatings) tend to adsorb fewer opsonins and circulate longer. By comparison, nominally neutral but hydrophobic surfaces still form robust coronas and can aggregate, leading to faster clearance. Thus, prolonged circulation is associated not with neutrality *per se*, but with near-neutrality plus surface hydration; note that apparent zeta potential is medium-dependent and can shift as the corona evolves (Tekie et al., 2020; Ernsting et al., 2013). Surface hydrophobicity has a similar strong influence: hydrophobic nanoparticles without protective coatings instantly adsorb a broad array of plasma proteins (IgG, complement factors, fibrinogen, *etc.*), forming a “hard” corona that flags them for phagocytic clearance. In contrast, hydrophilic or “stealth” coatings (such as PEGylation or biomimetic lipid membranes) greatly limit non-specific protein adsorption, yielding a thinner corona enriched in dysopsonins, benign proteins like apolipoproteins and clusterin that help the nanoparticle hide from the immune system (Aoyama et al., 2016). This stealth corona prolongs circulation stability but can reduce direct cellular uptake, meaning designers must balance stealth properties with targeted delivery mechanisms. Overall, tuning surface charge and hydrophobicity is crucial for optimizing pharmacokinetics: a moderately hydrophilic, slightly charged surface can minimize aggregation and opsonin tagging, extending the nanoparticle’s plasma half-life and increasing the likelihood of it reaching its intended site of action before clearance (Tekie et al., 2020; Ernsting et al., 2013; Aoyama et al., 2016; González-García et al., 2022).

The material composition and mechanical properties of nanoparticles also critically determine their *in vivo* fate and performance. The core composition (organic vs. inorganic) affects stability and biodegradation: non-degradable inorganic nanoparticles (e.g., gold, silica) tend to persist and accumulate in organs over time, as evidenced by gold particles still retained at high levels in liver and spleen months after injection (Jakic et al., 2024; Ontiveros‐Robles et al., 2023; Liu et al., 2025). By contrast, biodegradable polymer- or lipid-based nanoparticles (e.g., PLGA polymers, liposomes) can break down into minor metabolites that are excreted, preventing long-term buildup and allowing repeat dosing. Composition also influences the rigidity and interactions of the nanoparticle—for example, coating hard inorganic cores with a biomimetic lipid or polymer shell can make them more “soft” or deformable, aiding their transport through filtration mechanisms in the spleen and liver (Rompicherla et al., 2021). Nanoparticle elasticity (stiffness) has emerged as an essential factor linking physicochemical traits to pharmacokinetics (Bozzer et al., 2024). Softer, more deformable nanoparticles are better able to squeeze through biological barriers and avoid mechanical entrapment or immediate uptake by macrophages. In one study using hydrogel-based nanoparticles (∼120 nm), very soft particles (Young’s modulus ≈18 kPa) exhibited a blood circulation half-life of ∼20 h, roughly double that of much stiffer counterparts (∼1,350 kPa modulus, ∼9 h half-life) (Hui et al., 2019; Desai et al., 2022). The stiffer particles were cleared faster and sequestered more in filtering organs, whereas the softer ones circulated longer and achieved greater tissue distribution (Wang et al., 2025; Kong et al., 2021). Mechanistically, moderate elasticity was shown to favor the adsorption of specific “stealth” proteins; for instance, nanoparticles with intermediate stiffness (on the order of 10^2^–10^3^ kPa) preferentially accumulated apolipoprotein A-I in their corona, and this correlated strongly with extended circulation *in vivo* (Bozzer et al., 2024; Li M. et al., 2022; Zhang et al., 2024). By adjusting the material makeup (e.g., using flexible polymers or adding plasticizers) to tune stiffness, researchers can thus indirectly control protein corona composition and biodistribution profiles (Tekie et al., 2020). In summary, a nanoparticle’s inherent makeup—its composition and stiffness—will influence its degradation, its navigation through the body, and its ability to deliver drugs to target sites. Optimizing these physicochemical properties holistically is key to achieving favorable pharmacokinetics (prolonged, targeted circulation) and pharmacodynamics (effective drug action at the site of disease) for nanoparticle-based therapeutics. Table 1 synthesizes key mechanistic links between nanoparticle physicochemical traits and their pharmacological fate, integrating quantitative thresholds with observed biological outcomes.

**TABLE 1:** Mechanistic relationships between nanoparticle physicochemical properties and their Impact on protein corona formation, circulation stability, and biological transport.

Physicochemical property	Influence on protein corona and biofluid interactions	Influence on circulation stability	Influence on cellular and tissue transport	References
Size	Particle size strongly shapes protein-corona composition and load: ∼80-nm carriers typically adsorb fewer opsonins than larger (170–240 nm) counterparts, correlating with slower clearance and longer circulation.	Determines clearance route and half-life. <∼6 nm NPs are rapidly cleared renally, >200 nm are sequestered in the spleen and removed faster *via* MPS/RES. ∼100 nm often optimizes circulation, enhancing tumor EPR uptake while avoiding rapid kidney or immune clearance. Particles ranging from 10 to 100 nm tend to clear more slowly than those greater than 200 nm, thereby preventing sequestration in the spleen while avoiding renal filtration.	Facilitates barrier crossing and tissue distribution. Sub-100 nm NPs can extravasate into tumors (EPR effect) and, in some cases, cross the BBB. NPs with a diameter of 200 nm extravasate less, remaining mainly in circulation or lymph. Smaller NPs diffuse more easily through dense tissues, improving penetration.	Haripriyaa and Suthindhiran (2023), Buzea et al. (2007), Foroozandeh and Aziz (2018), Jeong et al. (2021)
Shape	Affects protein adsorption and corona makeup. Rod-shaped NPs absorb more plasma proteins than the same-size spheres, with shape-dependent shifts in bound proteins (e.g., immunoglobulin vs albumin), showing geometric shapes’ corona composition.	Affects hydrodynamics and immune recognition, altering circulation time. Non-spherical particles (rods, worms, discs) often circulate longer and evade macrophages better than spheres. High-aspect-ratio shapes slip past phagocytes, slowing clearance, while spheres are more readily engulfed.	Modulates margination, uptake, and organ distribution. Rods often lodge in the lungs, and disks in the spleen. Spheres are taken up mostly by mass, but rods/worms are in higher numbers. Non-spherical shapes generally have lower uptake and greater vessel margination, enhancing tumor deposition while reducing non-specific uptake.	Madathiparambil et al. (2020), Singh and Lillard (2009)
Surface Charge	Alters corona *via* electrostatic protein binding: cationic surfaces attract negatively charged (low pI) proteins, anionic surfaces attract positively charged (high pI) ones. IgG and albumin bind strongly to charged groups (e.g., –NH_2_, –COOH); excess IgG can trigger immune clearance, making charge tuning crucial.	Moderate surface charge (slight ζ±) improves stability and prolongs circulation by reducing aggregation and opsonization. Nominally neutral but hydrophobic surfaces can still opsonize/aggregate; near-neutral plus hydrophilic (‘stealth’) coatings tend to prolong circulation. Optimal charge helps evade early clearance.	Drives cell interaction and uptake. Cationic NPs bind negatively charged membranes, boosting uptake (proper for gene delivery). Anionic NPs have lower uptake but can enter *via* non-specific or scavenger pathways. Charge can be tuned to enhance targeting or limit non-specific uptake.	Florez et al. (2012), Wu et al. (2015), Sterin et al. (2025), Funk et al. (2022)
Composition (Material)	Material composition (organic vs. inorganic) shapes protein adsorption and bio-interactions. Hydrophobic polymer or metal-oxide NPs without hydrophilic coatings adsorb diverse serum proteins (large “hard” corona) and may activate complement. Biomimetic or lipid-based NPs (e.g., liposomes) attract apolipoproteins and fewer immunogenic proteins, yielding dysopsonin-rich coronas (e.g., ApoA1, clusterin) that help evade clearance, unlike bare inorganic particles that bind opsonins readily.	Determines *in vivo* stability and degradation, affecting circulation time. Inorganic NPs (e.g., gold, silica) are non-degradable and may accumulate over the long term. Biodegradable polymeric (PLGA) or lipid NPs break down into soluble units, enabling clearance and reducing organ buildup. Biocompatible types (e.g., liposomes) are biodegradable, non-toxic, and generally well-tolerated; they can trigger CARPA in susceptible settings, allowing repeated or prolonged use.	Influences distribution and targeting. Inorganic cores (e.g., iron oxide, gold) often accumulate in the liver/spleen, facilitating imaging but limiting deep-tissue delivery. Polymeric or lipid NPs enable target-responsive release and better tissue penetration *via* flexibility or small degradation products. Composition also affects stiffness and pore navigation. “Stealth” designs (e.g., cell membrane or hydrophilic polymer coatings) aid body-wide travel without early sequestration.	Mathaes et al. (2014)
Surface Hydrophobicity	Strongly affects protein binding: hydrophobic surfaces rapidly adsorb opsonins (IgGs, complement, fibrinogen), forming a corona that promotes immune recognition. Hydrophilic surfaces, especially PEGylated surfaces, resist nonspecific adsorption, yielding a thinner, more selective, or “stealth” corona enriched in inert proteins.	Key determinant of stealth vs. clearance. Hydrophobic NPs are rapidly opsonized and cleared. Hydrophilic surfaces (e.g., PEGylation) block protein binding, prolonging circulation, though excessive hydrophilicity may reduce target cell uptake, requiring a balance.	Affects uptake and biodistribution *via* corona and solubility. Hydrophobic NPs may insert into membranes or be endocytosed, but are usually protein-coated, dictating uptake. Hydrophilic/PEGylated NPs circulate broadly, accumulate *via* EPR, and often need targeting ligands for entry. Hydrophobic surfaces favor rapid clearance and local sequestration; hydrophilic surfaces favor wide distribution and longer extracellular presence.	Haripriyaa and Suthindhiran (2023), Mathaes et al. (2014)
Elasticity (Stiffness)	Regulates corona dynamics and composition. Softer vs. stiffer NPs recruit different proteins; intermediate elasticity (∼10^2^–10^3^ kPa) favors ApoA1 adsorption, linked to prolonged circulation. Very rigid materials may attract more opsonins, while ultra-soft materials may deform and expose new binding sites; elasticity thus tunes biological identity.	Impacts circulation by influencing mechanotransduction and phagocytosis. Very soft NPs can evade clearance by deforming through splenic filters, but if too soft, they may be taken up when spread. Very stiff NPs resist deformation but risk immune recognition or trapping. In mice, intermediate stiffness yielded the most extended half-life, likely *via* protective coronas (e.g., ApoA1) and reduced sequestration, suggesting that stiffness tuning can enhance stability.	Alters uptake and tissue penetration. Stiff NPs enter *via* classical endocytosis but penetrate dense tissue poorly. Soft NPs deform through barriers, improving tumor penetration and reducing phagocytosis, though excess flexibility may limit internalization. Elasticity thus tunes circulation behavior, cell interactions, and tissue infiltration.	Hui et al. (2019), Li et al. (2022a), Hui et al. (2018)

Abbreviations: NP/NPs, nanoparticle(s); MPS, mononuclear phagocyte system; RES, reticuloendothelial system; EPR, enhanced permeability and retention; BBB, blood–brain barrier; PEG, polyethylene glycol; PEGylated, polyethylene-glycol–modified; PLGA, poly (lactic-co-glycolic acid); IgG, immunoglobulin G; ApoA-I (often written ApoA1), apolipoprotein A-I; ζ-potential (zeta potential), electrokinetic surface potential.

In conclusion, the interplay between nanoparticle size, shape, surface chemistry, composition, and mechanical properties defines their biological identity and dictates how they behave *in vivo*. Each parameter influences protein corona formation, immune recognition, clearance pathways, and the ability to reach and act within target tissues, ultimately shaping both pharmacokinetics and pharmacodynamics. A holistic design strategy balancing these traits to achieve the desired stability, biodistribution, and cellular engagement offers the most effective route to creating nanomedicines with predictable, optimized therapeutic performance.

Table 2 (“Translational reality-check”) complements the mechanistic-to-metric map by converting mechanistic design principles into actionable CMC and early clinical checkpoints. It specifies the minimum pre-FIH evidence and controls for four recurrent bottlenecks (immune compatibility, lot-to-lot comparability, scalability/process control, and regulatory alignment). It pairs each with early clinical readouts and mitigations. In particular, the table emphasizes proactive screening and management of complement activation–related pseudoallergy and anti-PEG/ABC phenomena, orthogonal analytics with a mechanism-linked potency assay to anchor comparability, and validated manufacturing routes with defined design space and PAT to preserve performance at scale. Together, these controls operationalize the framework into regulatory-grade decision criteria. They enable PBPK-informed first-in-human dose selection, reduce PK/PD drift across scale-up and post-change, and pre-empt standard failure modes at re-dosing and in heterogeneous clinical settings, thereby de-risking translation while preserving mechanistic fidelity (Lin et al., 2015; Sindhwani et al., 2020).

**TABLE 2:** Translational reality-check for nanoparticle therapeutics: immune safety, lot-to-lot comparability, scalability, and regulatory alignment.

Translational domain	Anticipated risk/question to answer	Minimum pre-FIH evidence and CMC controls	Early-clinical readouts and mitigations	References
Immune responses and hemocompatibility (CARPA, anti-PEG/ABC)	Infusion reactions; complement activation; loss of exposure on re-dose (ABC); cytokine spikes	*In vitro*/*ex vivo* hemocompatibility (hemolysis, platelet activation), complement split products (C3a, C5a, sC5b-9), cytokine panel; endotoxin control; define specs for osmolality/pH; justify “stealth” strategy (PEG vs. zwitterionic)	Sentinel dosing; infusion-rate control; premedication where indicated; serial complement/cytokines; anti-PEG/anti-ligand serology pre/post re-dose.	Gabizon et al. (2003), Aoyama et al. (2016), Mathaes et al. (2014)
Lot-to-lot comparability (CQA definition)	PK/PD drift or immunogenicity due to subtle changes in size/PDI, ζ-potential, ligand density, drug loading/release	Define CQA panel: size/PDI (DLS/NTA), ζ-potential, morphology (TEM/cryo-EM), drug load/release kinetics, ligand density/activity, endotoxin/sterility; orthogonal analytics; mechanism-linked *potency assay* aligned with the mechanism-to-metric map.	Batch release tied to CQA acceptance + potency; trend analysis and predefined CAPA; bioequivalence bridging if process changes	Ernsting et al. (2013), Li et al. (2022a)
Scalability and process control	Loss of performance moving from bench to plant; drift in size/load; sterility failures.	Define manufacturing route (e.g., microfluidization/HPH → TFF → lyophilization); establish *design space*; in-process PAT (e.g., inline DLS/UV); validated aseptic strategy and 0.22 µm compatibility where applicable; robust reconstitution protocol.	Comparability plan covering CQA + potency + abbreviated PK *in vivo*; stress an engineering change log.	He et al. (2017), Homayouni et al. (2014)
Bioanalytical strategy (free vs. encapsulated)	Misleading PK if free/encapsulated drug not separated; artefacts from matrix effects	Orthogonal separation (SEC/ultrafiltration/FFF-MALS) to quantify free vs. encapsulated; validated assays for NP counts (NTA/ICP-MS for inorganics) and ligand density; stability in matrix (adsorption, degradation)	Report exposure for both total drug and pharmacologically relevant fraction; sensitivity analyses for handling artefacts	Li et al. (2022a), Lin et al. (2015)
PBPK-informed FIH dose selection	Over- or under-dosing due to non-intuitive NP disposition; species scaling pitfalls	Mechanistic PBPK parameterized with CQA-linked transport (MPS clearance, permeability, release); VandV against preclinical PK/BD; first-in-human starting dose justified by exposure margins	Sparse PK with particle-aware sampling windows; model-informed dose-escalation; prospective model updates	Yuan et al. (2019), Lin et al. (2015)
EPR heterogeneity and active transport.	Over-reliance on EPR; poor tumor delivery in some patients.	Preclinical evidence for route(s) of entry (intravital or equivalent); consider active mechanisms (endothelial transcytosis) or microenvironment modulation.	Imaging or circulating biomarkers of permeability/vascular targets; consider adjuncts (e.g., iRGD) in early studies	Sindhwani et al. (2020), Sugahara et al. (2010), Tylawsky et al. (2023)
Lymphatic routing (oral/SC) and excipient effects.	Unintended P-gp/CYP modulation; off-target immunomodulation; variable lymph uptake.	Justify lipid chain length/surfactants; demonstrate lymphatic uptake (mesenteric lymph, chylomicron association); DDI risk assessment if excipients modulate transporters/enzymes.	PK with lymphatic assessments when feasible; monitor DDI signals; titrate excipient load	Khan et al. (2016), Chaturvedi et al. (2020)
Material persistence and long-term safety.	Accumulation of non-degradable cores (liver/spleen); chronic toxicity.	Biodistribution/clearance (incl. mass balance) over extended windows; histopathology; design degradability or eliminate persistent fractions	Long-term safety labs/imaging where relevant; redosing rules based on accumulation	Jakic et al. (2024)
Mechanical properties (stiffness/elasticity).	Unintended shifts in corona, MPS uptake, and PK	Tie modulus to corona composition (ApoA-I enrichment) and circulation; specify modulus target and test window.	Monitor exposure vs. expected for modulus band; adjust if PK deviates.	Hui et al. (2019), Li et al. (2022a)
Device and administration compatibility.	Loss of dose (adsorption), particle destabilization in lines/filters.	Extractables/leachables risk; adsorption screens (bags/lines/filters); filterability (0.22 µm) vs. integrity	Administration SOPs (line material, filter spec, flush); infusion-profile controls	Tan et al. (1993)
Regulatory alignment (QbD/ICH) and change management.	Delays or rework due to unclear control strategy; post-change inequivalence.	QbD control strategy mapped to ICH Q8/Q9/Q10/Q12; predefined comparability protocol (CQA + potency + PK) for scale/site changes; stability (accelerated/long-term; agitation/light; cold chain).	Regulatory-grade documentation of design space, PAT, and comparability readouts; model-informed justification packages	(Yuan et al., 2019; ICH. ICH Harmonised Tripartite Guideline: Safety, 2000; ICHQ13, 2021; ICHS8. ICH Harmonised Tripartite Guideline, 2005)
Clinical endpoints and safety readouts.	PK-PD disconnect; missing early translational signals.	Prespecify NP-relevant safety markers (CARPA panel, ABC on re-dose) and PD metrics aligned to mechanism (from the mechanism-to-metric map); enrich cohorts by vascular/immune phenotype where justified.	Composite early endpoints: exposure (AUC, t½), target-tissue signals (imaging/biomarkers), immunologic safety; adaptive escalation	(Gabizon et al., 2003; Ernsting et al., 2013)

Abbreviations: ABC, accelerated blood clearance; CQA, critical quality attribute; CARPA, complement activation-related pseudoallergy; DDI, drug–drug interaction; DLS, dynamic light scattering; FFF-MALS, field-flow fractionation–multi-angle light scattering; FIH, first-in-human; HPH, high-pressure homogenization; ICH, international council for harmonisation; MPS, mononuclear phagocyte system; NTA, nanoparticle tracking analysis; PAT, process analytical technology; PBPK, physiologically based pharmacokinetics; PEG, polyethylene glycol; PK/PD, pharmacokinetics/pharmacodynamics; PLD, pegylated liposomal doxorubicin; SOP, standard operating procedure; TFF, tangential-flow filtration; ζ-potential, zeta potential.

## 4 Mechanistic modulation of pharmacokinetics

### 4.1 Absorption

NP systems can significantly enhance drug absorption by addressing solubility and biological barriers in the gastrointestinal tract (Li J. et al., 2022). Encapsulating poorly water-soluble compounds into nanoscale carriers enhances their dissolution rate and apparent solubility, thereby increasing uptake. For example, curcumin loaded in solid lipid nanoparticles (with P-gp inhibiting excipients like TPGS) achieved a 12.3-fold higher oral AUC compared to curcumin suspension (Ji et al., 2016). Such nanocarriers also bypass intestinal efflux pumps. Paclitaxel, a P-glycoprotein (P-gp) substrate with negligible oral bioavailability, showed a 2.4-fold increase in bioavailability when formulated in sustained-release lipid nanoparticles compared to the conventional formulation. The mechanism involves prolonged residence and uptake *via* the lymphatic pathway, which effectively avoids P-gp-mediated extrusion in gut enterocytes. Lymphatic absorption is a key route for extremely lipophilic drugs, such as paclitaxel and curcumin, when incorporated into long-chain lipid nanocarriers, which promote transport *via* intestinal chylomicrons, thereby bypassing first-pass extraction in the liver (Jeong et al., 2021; Chaturvedi et al., 2020). This strategy yields gains in systemic exposure; for instance, quercetin nanoparticles (3–5 nm) improved quercetin’s oral bioavailability by >5-fold (523% increase) relative to the free form (Chitkara et al., 2012; Chettupalli et al., 2025). These examples illustrate that nano-formulations can quantitatively enhance absorption by improving dissolution, preventing efflux, and utilizing alternative uptake pathways.

Beyond solubility and residence time, many nanoformulations also mitigate multidrug resistance (MDR) at the intestinal epithelium by reducing exposure to ATP-binding cassette (ABC) efflux, most notably P-gp/ABCB1. Carrier uptake *via* endocytosis and chylomicron-mediated lymphatic routing limits apical efflux and first-pass extraction, increasing enterocyte-to-lymph transfer for highly lipophilic substrates. Moreover, pharmacologically active excipients commonly used in nanocarriers, such as TPGS, act as P-gp modulators (inhibiting ATPase activity and altering membrane microviscosity), which further augments transcellular transport and net systemic entry. Together, these mechanisms raise intracellular residency and net flux even for classic P-gp substrates, explaining part of the multi-fold oral AUC gains observed with long-chain lipid/TPGS systems (Chaturvedi et al., 2020; Ji et al., 2016; Dilliard et al., 2021; Jia et al., 2025).

### 4.2 Distribution

Nanotechnology enables precise control over drug distribution, leveraging both passive and active targeting mechanisms (Salimi et al., 2024). In oncology, long-circulating nanoparticles capitalize on the Enhanced Permeability and Retention (EPR) effect to passively accumulate in tumor tissue. Tumor vasculature’s leakiness allows nanoscale carriers to extravasate and concentrate drugs at the tumor site, raising tumor-to-normal tissue drug ratios well above those of free drug. Moreover, decorating nanoparticle surfaces with targeting ligands can actively direct them to specific cells or receptors, compounding the benefits of EPR. In one striking example, a peptide-targeted liposomal doxorubicin (functionalized with an NSCLC-homing peptide) achieved 11.2-fold higher doxorubicin accumulation in tumors compared to free doxorubicin; consequently, the tumor AUC over 72 h was increased ∼159-fold (Wu et al., 2015; Chang et al., 2013). This demonstrates how active targeting can profoundly enhance localized drug delivery and retention.

Nanoparticles also enable drug access to privileged sites like the brain. Under normal conditions, the blood–brain barrier (BBB) severely restricts most drugs, but surface-modified NPs can traverse or circumvent this barrier. For instance, poly (butyl cyanoacrylate) nanoparticles coated with polysorbate 80 were shown to ferry doxorubicin into rat brains at concentrations >6 μg/g. In contrast, free doxorubicin remained undetectable (<0.1 μg/g). These results are model-dependent and not uniformly reproduced in humans; surfactant-mediated BBB passage raises concerns about safety and translatability (Gulyaev et al., 1999; Foglizzo and Marchiò, 2022). This represents an over 60-fold enhancement in brain drug levels due to the NP’s interaction with BBB transport mechanisms (Gulyaev et al., 1999). Such results highlight that by altering surface chemistry (e.g., using surfactants or targeting moieties), NPs can profoundly alter biodistribution, delivering drugs to previously inaccessible sanctuary sites.

Crucially, the size and surface properties of nanoparticles are engineered to modulate where the drug goes in the body. Particle size dictates extravasation and uptake: very small NPs (<∼5–6 nm) tend to undergo rapid renal clearance (minimal tissue retention), whereas moderately sized NPs (∼50–100 nm) circulate longer and deposit more in target tissues (Suk et al., 2016). A systematic study of 20, 50, and 200 nm drug–silica nanoconjugates found that 50 nm particles provided the best tumor retention and penetration, leading to superior efficacy, compared to both smaller and larger sizes (Tang et al., 2014). Surface “stealth” modifications also skew distribution profiles. For example, PEGylated (“stealth”) nanoparticles resist opsonization and uptake by the mononuclear phagocyte system, prolonging circulation and thereby enabling greater tumor accumulation *via* EPR (Suk et al., 2016; Chaturvedi et al., 2020; Tan et al., 1993). In sum, by tuning size (for optimal tissue penetration) and surface (for avoidance or targeting), nanocarriers achieve differential biodistribution, concentrating drugs in desired sites (tumors, brain, *etc.*) while sparing off-target organs.

### 4.3 Metabolism

Nanoparticle delivery can profoundly influence drug metabolism by shielding the payload from premature biotransformation and by modifying the route and rate at which the drug encounters metabolic enzymes. One advantage is protection from first-pass metabolism: when nanoformulations divert absorption from the portal blood to the lymphatic circulation, the drug effectively skips the hepatic first-pass filter (Chaturvedi et al., 2020; Tan et al., 1993). Lipid-based nanosystems with long-chain triglycerides exemplify this; they channel highly lipophilic drugs into intestinal lymphatics, significantly reducing initial hepatic extraction. Even beyond oral delivery, encapsulating a drug within a nanoparticle can sterically hinder enzymatic access until the carrier releases its payload, thereby prolonging the parent drug’s circulation time. At the tissue level, nanoparticles can also help overcome tumor cell MDR by altering intracellular trafficking (endocytosis → endo/lysosomal release → cytosol) and sustaining high local drug activity that transiently saturates efflux. Co-delivery strategies amplify this effect (e.g., embedding TPGS or related P-gp modulating lipids within the carrier shell, or pairing the payload with selective enzyme/transport modulators) to keep cytosolic concentrations above the efflux-controlled threshold and prolong active drug exposure (Chaturvedi et al., 2020; Ji et al., 2016; Paolini et al., 2017; Fin et al., 2025).

Another powerful approach is to incorporate metabolic enzyme inhibitors into nanocarriers to reduce drug metabolism actively. By co-delivering a metabolism modifier at the site of action, one can selectively inhibit enzymes that would otherwise deactivate the drug. A vivid demonstration comes from Paolini *et al.* (2017), who designed galactosamine-targeted PLGA nanoparticles carrying a natural CYP3A4 inhibitor and delivered them to hepatocytes (Paolini et al., 2017)—pretreating tumor-bearing mice with these nanoinhibitors before docetaxel treatment protected the drug from rapid hepatic metabolism. The result was a significantly enhanced antitumor effect: the nanoparticle strategy slowed tumor growth to 32% of the control (*versus* untreated) and improved survival from 0% to 67% at 55 days (Paolini et al., 2017). This example highlights how mechanistic modulation of metabolism, achieved through enzyme inhibition delivered by NPs, can yield quantifiable therapeutic benefits. More generally, nanocarriers mitigate metabolic loss through a combination of physical protection, altered absorption pathways, and smart co-therapy, thereby maintaining higher active drug levels for longer durations.

### 4.4 Elimination

Through nanotechnology, drug elimination profiles can be tuned to reduce clearance and even alter excretory routes. Free small-molecule drugs are often rapidly cleared by renal filtration or metabolic/biliary elimination, leading to short half-lives. Encapsulation in nanoparticles tends to decrease the drug’s clearance from the bloodstream. One reason is size exclusion: typical drug-loaded NPs are larger than the renal glomerular filtration cutoff (∼5–10 nm), so they largely avoid immediate urinary excretion (Suk et al., 2016; Adhipandito et al., 2021). Instead of being lost in urine, the nanoparticle-bound drug remains in circulation or is taken up by the liver and spleen (*via* phagocytic cells), shifting elimination toward the hepatobiliary route. This prolongs systemic exposure dramatically. For instance, PEGylated liposomal doxorubicin (Doxil®) has an effective plasma half-life of ∼2–3 days, *versus* only a few hours for free doxorubicin, and its week-long AUC is roughly 90 times higher than that of the free drug (Suk et al., 2016). Such a reduction in clearance not only increases efficacy (by sustaining therapeutic concentrations) but can also lower toxicity by preventing high peak levels. Additionally, surface PEGylation and other “stealth” features prevent rapid opsonin-mediated removal by the spleen and liver, further extending circulation time (Suk et al., 2016). On the other hand, tiny nanoparticles (or those designed to degrade into sub-5 nm fragments) can be intentionally harnessed for renal elimination when rapid clearance is desired (Villanueva-Flores et al., 2020). Thus, by controlling nanoparticle size, surface, and degradability, one can redirect how a drug is eliminated, either delaying clearance to boost exposure or accelerating clearance to minimize off-target effects (Lin et al., 2015). The ability to modulate clearance and excretory pathways is a crucial aspect of nanoparticle pharmacokinetics, ensuring that therapeutic molecules remain in the body long enough to be effective but are ultimately cleared in a controlled manner.

### 4.5 Mechanistic modulation of pharmacodynamics

Altering a drug’s PK can profoundly change the intensity and duration of its pharmacodynamic (PD) effects. For instance, extending the elimination half-life or reducing clearance maintains therapeutic drug levels for more extended periods, thereby prolonging the duration of effect. Nanoparticle delivery systems often achieve this by prolonging circulation time and limiting distribution volume, thereby sustaining drug presence at the target site (Gabizon et al., 2003). As a result, peak plasma concentrations may be lower (mitigating acute toxic effects), but the total exposure (area under the curve, AUC) is markedly increased. For example, pegylated liposomal doxorubicin exhibits an order-of-magnitude longer half-life and ∼90× higher plasma AUC than free doxorubicin at comparable dosing, while maintaining lower peaks (Gabizon et al., 2003). This higher AUC and sustained drug release can intensify overall therapeutic outcomes by keeping concentrations above the efficacy threshold for longer, even if the immediate peak effect is moderated. In short, modified PK profiles (e.g., long circulating nanocarriers) tend to trade a lower instantaneous intensity for a more durable and controlled impact.

Changes in PK can also alter the concentration–response (exposure–effect) relationship. With conventional dosing, plasma drug concentration is a direct driver of response, but targeted or sustained-release formulations partially decouple this relationship. Enhanced delivery to specific tissues means the effective concentration at the site of action can be higher relative to plasma levels, shifting the dose–response curve. In practice, a nanoformulation may achieve the same or greater response at lower systemic concentrations because more of the drug reaches and remains in the target compartment (Gabizon et al., 2003). Quantitatively, at a matched dose, tumor growth inhibition improved from ∼57% with free doxorubicin to ∼78% with a nanoparticle formulation, illustrating how increased exposure translates into a greater antitumor effect (Alrohaimi et al., 2025). Moreover, the slow release or targeting can flatten the steepness of the concentration–response curve for off-target effects while maintaining steep efficacy in target tissues. This is evidenced by cases in which the nanoformulated drug requires a different dose to elicit maximal response: for example, a folate-targeted nanoparticle carrying methotrexate achieved full therapeutic effect in arthritis at a dose many-fold higher (in absolute terms) than that required for the free drug, yet with no added toxicity (Thomas et al., 2011). The free drug, by contrast, needed to approach its toxicity limit to reach the same anti-disease effect (Thomas et al., 2011). Such scenarios demonstrate that the traditional plasma concentration–response relationship is modulated by nanotherapy, effectively delivering higher concentrations to diseased cells at a given plasma level, resulting in a greater therapeutic response without a corresponding increase in systemic toxicity.

Perhaps the most crucial impact of PK modulation is on the drug’s therapeutic index (TI), the ratio of the toxic to the effective dose. By altering drug distribution and release, nano-delivery systems can widen the therapeutic index, making therapy safer and more effective. A classic goal of nanomedicine is precisely this: to improve efficacy while reducing toxicity. For example, pegylated liposomal doxorubicin was designed to enhance the therapeutic index of doxorubicin by increasing tumor-specific delivery and reducing cardiac exposure (Gabizon et al., 2003). Generally, a slower-releasing or targeted carrier will lower peak concentrations in vulnerable organs and concentrate the drug in disease tissues, thus raising the maximum tolerated dose or allowing higher cumulative dosing (Gabizon et al., 2003). In parallel, the minimum effective dose may decrease if more of the drug reaches the target. The result is a larger safety window. Quantitatively, improvements can be striking: a nanoparticle formulation can achieve the same therapeutic effect as the free drug at a fraction of the systemic exposure, or conversely, tolerate a much higher dose before toxicity. Both aspects effectively increase the TI (Thomas et al., 2011). Together with the PLD example above, these data provide concise quantitative anchors linking PK shifts (↑t½, ↑AUC) to PD gains and a widened therapeutic index (Gabizon et al., 2003; Alrohaimi et al., 2025; O’Brien et al., 2004; Rafiyath et al., 2012).

### 4.6 Case study: free vs. nanoformulated drug, exposure and effect relationship

A direct comparison of a free drug *versus* a nanoformulation highlights these principles. Doxorubicin (DOX), an anti-cancer agent, provides a clear case: in free form, it distributes widely and is cleared relatively quickly, whereas in a PEGylated liposomal form, it remains in circulation much longer and localizes to tumors. The liposomal DOX (e.g., Doxil®) exhibits an elimination half-life on the order of 20–30 h (vs. only ∼2 h for free DOX) and a plasma AUC dozens of times greater than the free drug (Gabizon et al., 2003). This increased exposure leads to significantly greater drug accumulation in tumor tissue (Gabizon et al., 2003). Correspondingly, the anti-tumor effect is enhanced: in murine models, a DOX-loaded lipid nanoparticle achieved about 78% tumor growth inhibition, compared with ∼57% with the same dose of free DOX (Alrohaim et al., 2025). The duration of tumor suppression is extended by sustained release and retention of DOX in the tumor interstitium. Equally important, toxicity outcomes diverge. Free doxorubicin is notorious for dose-limiting cardiotoxicity, whereas the nano-encapsulated form drastically reduces heart exposure. In the cited study, systemic toxicity (e.g., cardiac damage and nephrotoxicity) was significantly lower in the nanoparticle-treated group than in the free DOX group (Alrohaim et al., 2025). Clinically, this has allowed pegylated liposomal DOX to be given in higher cumulative doses than free DOX, since cardiac side effects are attenuated (Gabizon et al., 2003). The trade-off is that liposomal DOX can cause some new dose-limited toxicities (such as mucositis/hand-foot syndrome). Still, these are generally more manageable and occur at higher dose thresholds than free drug’s toxicities (Gabizon et al., 2003). Overall, this example demonstrates that by modulating PK (prolonging circulation, targeting tumor tissue, and controlling release), one can enhance the drug’s effect on the tumor while minimizing harm to normal organs, effectively shifting the exposure–response balance in favor of efficacy. Similar benefits have been observed in non-cancer settings. For instance, a folate-targeted nanoconjugate of methotrexate in an arthritis model delivered the drug specifically to inflamed joint macrophages, yielding complete disease suppression at doses 7.5-fold higher than the free drug’s tolerated dose with no systemic toxicity (Thomas et al., 2011). In that case, the nanocarrier’s altered PK (targeted delivery and prolonged residence in diseased tissue) not only maximized the anti-inflammatory response but also avoided the off-target effects (e.g., weight loss, organ damage) observed with free methotrexate (Bozzer et al., 2024; Foglizzo and Marchiò, 2022; Thomas et al., 2011). These comparative outcomes highlight how mechanistic modulation of PK translates into tangible PD advantages, including greater intensity and persistence of therapeutic effect, as well as a broader therapeutic index, thereby contributing valuable improvements to the state of the art in drug therapy.

### 4.7 Cross-compound pattern analysis

To identify universal patterns, we compiled a cross-comparison of 8 representative molecules (both natural products and synthetic drugs) with reported quantitative PK and PD data before and after nano-formulation. Table 3 summarizes key properties (lipophilicity and solubility), nano-carrier type, and the changes in PK (e.g., bioavailability or AUC) and PD (efficacy/toxicity outcomes) upon nano-delivery.

**TABLE 3:** Examples of molecules (natural and synthetic) show significant PK improvements and pharmacodynamic (PD) benefits upon nano-formulation.

Molecule (type, indication)	Physicochemical profile (logP; water solubility)	Nano-formulation	PK improvement (vs free)	Notable PD/efficacy changes	Comparator/confounders (flag and notes)	References
Curcumin (Natural polyphenol, anti-inflammatory)	∼3; <0.1 μg/mL (practically insoluble)	Colloidal dispersion (Theracurmin®)	Oral AUC ↑ ∼27× in humans (∼40× in rats)	Enabled clinical efficacy at lower dose vs unformulated curcumin (e.g., improved liver and inflammation markers).	Comparator likely unmatched (powder vs colloidal dispersion); possible vehicle/surfactant contribution to absorption (trend, not universal).	(Sasaki et al., 2011; Morimoto et al., 2013)
Resveratrol (Natural stilbene, antioxidant/anti-inflammatory)	∼3.1; ∼30 μg/mL (poorly soluble)	Surface-modified SLN (TMC–PA coated)	Multi-fold gains reported (e.g., ∼3.8× in mice), magnitude depends on formulation/route/comparator.	Enhanced anti-inflammatory effects (greater suppression of TNF-α, IL-6, *etc.*, in treated animals).	Surface modifiers (e.g., TMC) can act as permeability enhancers—potential vehicle effect; verify comparator matching.	(Intagliata et al., 2019; Ramalingam and Ko, 2016; Zhou et al., 2017)
Puerarin (Natural isoflavone, anti-inflammatory)	Low lipophilicity; poor permeability (BCS IV; <3% oral F; sol ∼2.6 g/L)	Polymeric nanoparticle (PLGA matrix)	Oral AUC ↑ ∼5× in rats (relative bioavailability)	Significantly greater anti-inflammatory efficacy: e.g., ∼5× lower dose PU-NP achieved similar or superior edema reduction vs free drug.	Check BCS/solubility consistency; ensure vehicle/excipient parity in control.	(Ahirrao et al., 2024; Xie et al., 2013; Beg et al., 2022)
Celastrol (Natural triterpenoid, immunomodulator)	5.6; ∼11 μg/mL (very low solubility)	Silk-fibroin nanoparticle (IV; also oral forms studied)	IV AUC ↑ 2.4-fold; Oral absolute F ↑ from ∼3.1% to 7.6% (≈2.4×)	Improved therapeutic effect in disease models (enhanced anti-tumor and anti-inflammatory activity due to sustained exposure).	No explicit efflux-inhibiting excipients reported in cited IV study; oral comparisons may involve vehicle differences—interpret as formulation-dependent.	(Guo et al., 2021; Onyeabor et al., 2019; Zhan et al., 2020)
Celecoxib (Synthetic NSAID, COX-2 inhibitor for arthritis)	3.5; 1–3 μg/mL (BCS II, poorly soluble)	Nanosuspension (nanocrystal, TPGS-stabilized)	Oral AUC ↑ ∼2.5-FOLD in rats (245.8% ↑ in relative bioavailability)	More consistent absorption and higher plasma levels, potentially improving pain/inflammation control in osteoarthritis/rheumatoid arthritis (reducing variability vs oral capsule).	Confounder: TPGS (P-gp inhibition/solubilization). Comparator likely mismatched (capsule vs TPGS-stabilized nanosuspension). Do not ascribe full effect to “NP” alone.	(He et al., 2017; Homayouni et al., 2014)
Tacrolimus (Synthetic macrolide, immunosuppressant)	∼3; <10 μg/mL (BCS II, poor solubility; extensive first-pass)	Nanostructured lipid carriers (oral)	Oral AUC ↑ ∼7.2-fold in rats (relative bioavailability)	∼19× higher lymphatic uptake vs suspension promotes targeted immunosuppression (improved drug delivery to lymphoid tissues) and may reduce systemic toxicity.	Likely long-chain lipids/surfactants promote lymphatic transport; verify if comparator matches vehicle. Part of effect may reflect vehicle-driven lymphatic uptake.	Khan et al. (2016)
Docetaxel (Synthetic anticancer taxane)	∼3.2; <10 μg/mL (highly hydrophobic)	PEGylated polymer nanoparticle (IV)	IV AUC ↑ ∼38-fold; t1/2 prolonged ∼5.2-fold in mice	Enhanced tumor uptake and efficacy with reduced systemic toxicity (NP group showed greater tumor drug levels and lower side-effects than free DTX).	Comparator vehicle differs (e.g., polysorbate-based solutions common for free DTX); toxicity/PK differences may partly reflect vehicle rather than NP architecture alone.	(Ernsting et al., 2012; Guo et al., 2021)

Abbreviations: NP, nanoparticle; PK, pharmacokinetics; PD, pharmacodynamics; AUC, area under the plasma concentration–time curve; F, oral bioavailability; t1/2, terminal half-life; IV, intravenous; SLN, solid lipid nanoparticles; PLGA, poly (lactic-co-glycolic acid); TPGS, D-α-tocopheryl polyethylene glycol 1000 succinate; DTX, docetaxel; BCS, biopharmaceutics classification system; NSAID, nonsteroidal anti-inflammatory drug; COX-2, cyclooxygenase-2; TNF-α, tumor necrosis factor-alpha; IL-6, interleukin-6; PEGylated, polyethylene-glycol–modified; TMC–PA, denotes a TMC (N,N,N-trimethyl chitosan)–based surface modification.

Examining the examples in Table 3 reveals several general patterns in how nano-carriers influence drug PK/PD.

#### 4.7.1 Increment in exposure for poorly soluble drugs

Across heterogeneous reports, many, though not all, compounds with moderate-to-high lipophilicity (logP ≳ 3) and low aqueous solubility (<∼10 μg/mL) exhibit increases in systemic exposure following nanoformulation, with the magnitude of gain typically being multi-fold and contingent on the formulation, route, and comparator (Khan et al., 2016; Sasaki et al., 2011). For example, in one study, curcumin (logP ∼3, water-insoluble) achieved a 27-fold higher AUC when delivered as a colloidal nanoparticle dispersion compared with as a powder (Sasaki et al., 2011). Tacrolimus and celastrol, both extremely hydrophobic, have shown AUC increases of ∼5–7-fold with lipid-based nanocarriers under specific formulations and study conditions (Khan et al., 2016). Importantly, part of the observed gain in some entries is attributable to vehicle/excipient effects (e.g., TPGS or surfactants) and comparator mismatch, as flagged in Table 3, so these examples illustrate an indicative trend, not a universal rule.

#### 4.7.2 Enhanced absorption and reduced PK variability

Nanoparticle delivery improves the *rate and extent* of absorption for BCS class II/IV drugs. In the case of celecoxib (logP 3.5, sol. ∼2 μg/mL), reducing particle size to the nano-scale increased dissolution rate and intestinal uptake, yielding a ∼2.5-fold higher AUC and C_max_ *in vivo* (He et al., 2017). However, TPGS-stabilized nanosuspensions introduce a confounder (TPGS can modulate P-gp and solubilization), and in some studies, comparator vehicles differ, so improvements should be interpreted as NP + vehicle where applicable. More advanced nano-carriers can push this further, e.g., a silica-lipid hybrid microparticle achieved ∼6.5-fold AUC boost for celecoxib in dogs (He et al., 2017). Another benefit is more consistent plasma levels: nano-formulations bypass erratic absorption phases, smoothing out PK profiles (He et al., 2017). This consistency can be critical for drugs with narrow therapeutic windows or those prone to food-effect variability.

#### 4.7.3 Prolonged circulation and targeted delivery

Many nanocarriers (especially IV long-circulating formulations) significantly extend the drug’s half-life and retention in the bloodstream (Ernsting et al., 2012). Because free docetaxel is often formulated in polysorbate-based vehicles, part of the PK/toxicity contrast may reflect differences in the cars; Table 3 notes this caveat. Prolonged exposure can translate into greater drug accumulation in target tissues (tumors, inflamed sites) *via* enhanced permeability and retention (EPR) or lymphatic targeting (Bozzer et al., 2024; Ernsting et al., 2012). In tacrolimus NLCs, the nanoparticle’s affinity for lymphatic uptake resulted in ∼19-fold higher lymph delivery (Khan et al., 2016), although long-chain lipids/surfactants may contribute to lymphatic routing; see Table 3.

#### 4.7.4 Improved pharmacodynamic outcomes

The PD benefits of nano-formulation are evident across anti-inflammatory and other indications. Thanks to higher bioavailability and sustained levels, nano-delivered drugs often achieve greater efficacy at equal or lower doses. In a rodent inflammation model, puerarin-loaded NPs not only raised AUC ∼5×, but also drastically enhanced anti-inflammatory effects; a 12.5 mg/kg NP dose suppressed paw edema significantly more than the free drug, even matching the effect of an NSAID (indomethacin) (Ahirrao et al., 2024). Similarly, curcumin nanoparticles, by overcoming curcumin’s low absorption, have shown clinical efficacy in humans (improving disease biomarkers) at doses at which plain curcumin was ineffective (Sasaki et al., 2011; Morimoto et al., 2013). These PD gains are formulation- and model-dependent and, where vehicles differ or include active excipients, reflect the combined influence of NP architecture and vehicle (Miao et al., 2024). In general, inflammation and autoimmune disease models respond better to nano-formulated therapeutics, as higher drug exposure at the site of inflammation leads to more potent suppression of cytokines and inflammatory mediators (Intagliata et al., 2019; Salla et al., 2024; Debnath et al., 2023).

#### 4.7.5 Enhanced therapeutic index

Nanocarriers can also mitigate toxicity while boosting efficacy. The targeted and controlled release provided by NPs often reduces peak systemic concentrations and off-target exposure. For example, nanoencapsulated docetaxel showed increased tumor uptake with lower bone marrow toxicity than free docetaxel (Rafiei and Haddadi, 2017). Liposomal amphotericin B (a lipid NP formulation) is known to deliver the drug to fungal cells while sparing host tissues, drastically reducing nephrotoxicity (a classic example in antifungal therapy, though not an anti-inflammatory agent). Across compounds, improvements in therapeutic index should be interpreted in the context of the vehicle and comparator (as indicated in Table 2).

#### 4.7.6 Consistent trend across diverse compounds

The above patterns hold not only for anti-inflammatories but broadly for poorly soluble actives, with qualitative consistency rather than fixed quantitative thresholds. Whether the context is inflammation (curcumin, resveratrol, NSAIDs), immunosuppression (tacrolimus, cyclosporine), or oncology (taxanes, celastrol), the nano-formulation strategy frequently yields multi-fold increases in bioavailability/AUC, extended half-life, and enhanced efficacy (Sasaki et al., 2011; Ramalingam and Ko, 2016). Nonetheless, Table 3 emphasizes the use of comparator matching and excipient flags to avoid over-attributing effects to NP architecture alone. Researchers have noted that even for compounds already formulated in advanced solubilizing systems, nanoparticle approaches can surpass them. For example, cyclosporine A in a novel cubosome NP achieved ∼178% of the exposure of the already-optimized commercial microemulsion (Neoral®) (Wu et al., 2015), indicating further room to improve therapeutic delivery *via* nanotechnology.

In summary, evidence across heterogeneous studies indicates a recurring, yet context-dependent trend: nanocarriers are often most beneficial for compounds with poor water solubility and/or permeability, and they frequently yield multi-fold improvements in pharmacokinetic performance, with the magnitude contingent on formulation, route, and comparator rather than universal order-of-magnitude shifts. These PK gains can enhance pharmacodynamic outcomes in relevant models, particularly for anti-inflammatory and autoimmune indications, though effects are model- and formulation-dependent. The indicative heuristic emerging from this synthesis is that a drug with logP > 3 and solubility < 10 μg/mL is a plausible candidate for nano-formulation; such candidates may achieve multi-fold increases in AUC, and in specific settings effects can approach ≥5-fold with a lipid or polymeric nanoparticle system (Khan et al., 2016; Sasaki et al., 2011); however, part of the observed gains in some studies may reflect vehicle/excipient effects (e.g., TPGS, surfactants) and comparator mismatch, as flagged in Table 3. Consequently, nanoparticle delivery can be a pragmatic strategy to improve developability, mitigating solubility- and permeability-limited exposure and stabilizing PK profiles. The aligned yet heterogeneous data across compounds support this qualitative insight and provide indicative ranges, not fixed thresholds, for selecting nanocarrier approaches in development.

The classical enhanced permeability and retention (EPR) effect, wherein nano-sized drug carriers passively leak into tumors through fenestrated vasculature, has been a cornerstone of cancer nanomedicine. However, the assumption that “nano = EPR” is an oversimplification that has not translated into proportional clinical success. In practice, tumor accumulation of systemically injected nanoparticles is often minimal (on the order of only ∼0.5–1% of the injected dose) (Sindhwani et al., 2020), and the heterogeneity of tumor biology means EPR-driven delivery varies greatly between tumor types and patients. Detailed intravital studies have shown that the traditional model of nanoparticles diffusing through inter-endothelial gaps is largely *inaccurate*: up to ∼97% of nanoparticles were found to enter tumors *via* active processes through endothelial cells rather than *via* passive leaky gaps (Sindhwani et al., 2020). This fundamentally challenges the reductionist view that merely making a drug nanometer-sized guarantees effective tumor targeting.

To move beyond the EPR paradigm, active delivery mechanisms are being integrated into nanoparticle design. One strategy is receptor-mediated translocation, in which nanoparticles are engineered with ligands that bind to specific receptors on tumor cells or the vasculature, triggering active uptake or transport. For example, nanoparticles functionalized with fucoidan to target P-selectin on tumor endothelium were shown to induce caveolin-1-dependent transcytosis, actively ferrying the nanocarriers across blood vessel walls into the tumor tissue (Tylawsky et al., 2023). Such receptor-targeted approaches can “invite” nanoparticles into tumors rather than relying on random leakage, potentially overcoming the variability of passive EPR. Another approach is modulation of the tumor microenvironment to facilitate nanoparticle penetration. This includes strategies to transiently enhance vascular permeability or remodel physical barriers in tumor tissue. For instance, the *iRGD* tumor-penetrating peptide can increase vascular and tissue permeability in a tumor-specific, neuropilin-1-dependent manner, thereby allowing co-administered drugs or nanocarriers to penetrate deeper into the tumor interstitium (Sugahara et al., 2010). Likewise, alleviating the dense tumor stroma has proven beneficial: administering an antifibrotic agent (losartan) to reduce collagen in desmoplastic tumors significantly improved the intratumoral distribution and efficacy of subsequently delivered 100-nm liposomal doxorubicin (Doxil) (Diop-Frimpong et al., 2011). These examples illustrate that actively tweaking the tumor microenvironment, whether by biochemical or physical means, can augment nanoparticle delivery beyond what passive EPR alone would achieve.

Finally, interaction with the immune system is a critical Frontier beyond passive EPR. The mononuclear phagocyte system can rapidly clear nanoparticles, but immune cells can also be harnessed as allies for delivery. Emerging research shows that tumor-associated macrophages (TAMs) can actively mediate nanoparticle transport inside tumors: after nanoparticles extravasate, TAMs migrate toward them, engulf them, and carry them deeper into the tumor tissue, effectively redistributing the payload within the tumor microenvironment (Lin et al., 2022). This “Trojan horse” behavior of immune cells is now being explored as a delivery strategy, for example, loading therapeutic nanoparticles into circulating monocytes or neutrophils that naturally home to tumors, thereby ferrying drugs to regions that free nanoparticles might not efficiently reach. Integrating such immune-mediated delivery or avoiding immune sequestration (*via* stealth coatings or CD47-mimicking ligands) represents another layer of design to ensure nanocarriers reach their targets.

In summary, a critical re-evaluation of the EPR-centric mindset is underway. The next-generation of cancer nanomedicines is focusing on multifaceted targeting mechanisms that combine passive and active strategies to overcome the limitations of the EPR effect. By engaging specific cellular pathways, dynamically altering the tumor microenvironment, and co-opting the immune system, researchers aim to achieve more uniform and effective nanoparticle deposition in tumors (Sindhwani et al., 2020). This paradigm shift from a purely passive “EPR effect” model toward an actively steered delivery framework is expected to improve therapeutic outcomes and bridge the gap between promising preclinical nanomedicine and real-world clinical impact.

### 4.8 Standardized pharmacokinetic effects of nanoparticle formulations

As summarized in Table 4, nanoparticle (NP) formulations consistently exhibit superior pharmacokinetic profiles compared to conventional drug forms across diverse compounds. All reported AUC effect values are positive, indicating higher systemic exposure with NPs in every case, with increases ranging from modest (∼0.9, i.e., ∼2.5-fold for celecoxib) to very large (4.5, ∼90-fold for doxorubicin). Orally administered NPs of poorly bioavailable compounds show especially pronounced gains in absorption: for example, a colloidal curcumin formulation (Theracurmin®) achieved a ∼27-fold higher AUC in humans than an equivalent dose of curcumin powder (Nakagawa et al., 2014), and nanoencapsulated resveratrol, puerarin, celecoxib, and tacrolimus each yielded multi-fold AUC enhancements relative to suspensions or capsules. In the case of tacrolimus, incorporating the drug into a lipid nanostructured carrier not only boosted oral bioavailability by ∼7-fold but also increased lymphatic drug uptake by ∼19-fold, thereby effectively bypassing first-pass metabolism (Khan et al., 2016). For intravenous therapies, nanocarriers likewise conferred dramatic improvements: a PEGylated polymeric NP of docetaxel extended the drug’s half-life by roughly 5-fold and elevated AUC ∼38-fold compared to the conventional Taxotere® formulation. In contrast, PEGylated liposomal doxorubicin (Doxil®) achieved an order-of-magnitude higher AUC and markedly prolonged circulation (terminal t_1/2_) on the order of 2–3 days, *versus* only a few hours for free doxorubicin (Liu et al., 2024). Consistently, across all data points, the NPs showed reduced clearance (CL), commensurate with their higher exposure and longer circulation persistence. By the numbers (route-stratified). Across our eight-compound dataset, the median ln (AUC) effect for oral nanoformulations is 1.61 (≈5.0-fold; IQR ≈ 3–14×; range ≈ 2.5–27×), while for intravenous nanoformulations it is 3.64 (≈38×; range ≈ 2.4–90×). These values, while subject to vehicle/excipient confounding noted in Table 4, provide quantitative anchors for typical magnitude of NP-driven exposure gains at each route and are directionally consistent with field-wide evidence that stealth and pseudo-stealth strategies prolong circulation and elevate systemic exposure. For context and mechanisms underlying these effects, see recent syntheses on stealth carriers and PK control in nanomedicine (Wen et al., 2023; Li and Kataoka, 2021; Cabral et al., 2023).

**TABLE 4:** Standardized NP effects by route and vehicle (in-ratios of PK metrics).

Drug	Route	Species	Vehicle matched?	Dose (mg·kg^-1^)	AUC effect (ln-ratio)	Half-life effect (ln-ratio)	Clearance reduction (ln-ratio)	n (NP/Control)	SE/SD given?	95% CI	Notes (formulation vs. control; confounders)	Reference
Curcumin	Oral	Human	No	∼0.5 (30 mg)	3.3	NR	NR	12/12 (crossover)	Yes	NR	Colloidal dispersion (Theracurmin®) vs. powder (unmatched vehicle) – huge ↑AUC	​
Resveratrol	Oral	Mouse	No	∼50	1.34	—*(∼1.57)*	NR	6/6	Yes	NR	SLN (TMC–PA coated) vs. suspension (TMC polymer enhances mucosal absorption; vehicle differs)	​
Puerarin	Oral	Rat	No	12.5	1.61	NR	NR	6/6	Yes	NR	PLGA nanoparticle vs. suspension (BCS IV poor-permeability drug; ensure vehicle parity)	​
Celastrol	IV	Rat	No	1	0.88	NR	0.88	5/5	Yes	NR	Silk-fibroin NP vs. PEG 300 solution (no P-gp inhibitors; improved AUC, prolonged MRT)	​
Celecoxib	Oral	Rat	No	∼20	0.9	NR	NR	6/6	Yes	NR	TPGS-stabilized nanocrystal vs. capsule (TPGS excipient in NP; comparator not vehicle-matched)	​
Tacrolimus	Oral	Rat	No	∼1	1.97	NR	NR	6/6	Yes	NR	Lipid NLC vs. suspension (long-chain lipids in NP promote ∼19× lymphatic uptake; vehicles differ)	​
Docetaxel	IV	Mouse	No	40	3.64	1.64	∼0.03	4/4	Yes	NR	PEGylated polymer NP vs. Taxotere® (polysorbate/ethanol vehicle in free formulation; massive ↑AUC and t½, vehicle confounder)	​
Doxorubicin	IV	Human	No	∼1.5 (≈60 mg/m^2^)	4.5	∼3.00	4.5	∼6/6	Yes	NR	PEGylated liposomal DOX (Doxil®) vs. free DOX (aqueous solution); dramatically prolonged circulation (t½ ∼2–3 days vs. ∼2–4 h) and ≈90× AUC	​

Positive values denote nanoparticle (NP) benefit. AUC, effect = natural-log ratio of AUC (NP over control), Half-life effect, natural-log ratio of t½ (NP over control), and Clearance reduction = natural-log ratio of clearance (control over NP), so that higher (positive) values consistently indicate lower clearance with NP. Drug: index compound. Route: administration route (IV or Oral). Species: experimental model (mouse, rat, human). Vehicle matched? Yes, when NP and control share the same bulk excipients/vehicle (minus the nanoparticulate assembly); No, when vehicles differ or include active excipients (e.g., TPGS, polysorbates) that may confound PK, effects should be read as upper bounds of the NP, contribution; Unclear if insufficiently reported. Dose (mg·kg^-1^): nominal dose; for humans, per-kg approximations from labeled mg·m^-2^ are indicated where applicable. n (NP/Control): number of biological units contributing valid PK, data per arm; serial samples within a subject do not increase n; in crossover studies, the total participants are shown, and analyses are within-subject. SE/SD, given? Whether dispersion was reported and used to estimate 95% CI for ln-ratios; 95% CI, is omitted when variance is unavailable. Notes: formulation/comparator details and potential confounders (e.g., fed/fasted, use of P-gp inhibitors, digitized data). Reference: primary source. Symbols: “NR”, not reported; “∼”, approximate. Abbreviations: AUC, area under the concentration–time curve; t½, half-life; CL, clearance; NP, nanoparticle; SLN, solid-lipid nanoparticle; NLC, nanostructured lipid carrier; TPGS, d-α-tocopheryl polyethylene glycol succinate.

It should be noted that none of these studies employed perfectly vehicle-matched controls, meaning the NP formulations often included specialized excipients or matrices absent in the comparator (e.g., surfactants like TPGS in nanocrystals, lipid matrices in NLCs, or polymer–PEG systems in drug nanocarriers). This lack of vehicle matching introduces potential confounders–some of the observed pharmacokinetic advantages may partly reflect differences in formulation (solubilization aids, absorption enhancers, *etc.*) rather than the nanoscale features alone. For instance, the massive increase in curcumin exposure with Theracurmin® can be partially attributed to its colloidal dispersion and stabilizers relative to a raw powder dose, and the superior lymphatic uptake of tacrolimus with a lipid NP is facilitated by long-chain lipids that actively promote lymph transport (Khan et al., 2016). Therefore, the magnitudes of improvement reported in Table 4 represent *upper bounds* of the nanoparticle effect under those experimental conditions. Despite these caveats, the overarching trend is clear: NP-based delivery markedly enhances systemic exposure and prolongs drug half-life across a spectrum of drugs and models. The positive ln‐ratio values (for AUC and t_1/2_) and corresponding lower clearance values underscore the potential of nanotechnology to overcome bioavailability and rapid clearance limitations. Future studies with vehicle-matched controls will be valuable for quantitatively isolating the nanoparticle’s contribution. Still, the compiled evidence to date robustly supports the conclusion that nanoparticle formulations can substantially improve *in vivo* pharmacokinetic performance.

## 5 Conclusions and future perspectives

This review consolidates mechanistic principles governing how nanoparticle physicochemical properties, carrier type, and administration route reshape pharmacokinetics and pharmacodynamics. Size, shape, surface chemistry, composition, and elasticity collectively influence protein corona formation, immune recognition and clearance, and tissue delivery, thereby modulating exposure profiles, therapeutic intensity, and safety margins. We offer an evidence-informed framework with design heuristics and indicative ranges (rather than fixed thresholds) to anticipate PK/PD shifts and prioritize nanoformulation choices before undertaking resource-intensive *in vivo* work. The framework explicitly considers comparator matching and contributions from vehicle/excipients (e.g., TPGS, surfactants) to avoid over-attributing effects to nanoparticle architecture alone. It presents PBPK-informed considerations as hypothesis-generating, pending external validation. Beyond preclinical mechanisms, we also foreground translational needs, immune safety, batch-to-batch comparability, scalable manufacturing, and regulatory alignment to help bridge study design with CMC planning. Together, these components provide a compact mechanism-to-metric map that links nanoparticle traits to pharmacological performance and to recurrent translational risks.

Rather than reiterating all operational details discussed in previous sections, we emphasize several forward-looking priorities. A critical next step is the conduct of standardized, mechanistically oriented comparative studies with matched vehicles/vehicle-matched controls, harmonized endpoints, and transparent nanoparticle characterization (size/PDI, ζ-potential, morphology, and elasticity). Incorporating confounder flags, species/route stratification, and semiquantitative synthesis across subgroups will sharpen generalizability and refine and validate the indicative PK/PD ranges summarized here. Quantitative safety and translational metrics (e.g., CARPA, accelerated blood clearance, anti-PEG responses, long-term retention of inorganics, and EPR variability in humans) should be routinely integrated alongside efficacy. In parallel, integrating this mechanism-based framework with artificial-intelligence and machine-learning approaches (ranging from *in silico* nanoformulation screening to hybrid PBPK/ML “digital twin” models) could enable rapid exploration of large design spaces, learn structure–exposure–response relationships, and propose nanoformulations that satisfy multiple pharmacological and manufacturability constraints before *in vivo* testing. In parallel, clinical translation benefits from prospective assessment and mitigation of immune responses (e.g., CARPA, anti-PEG/ABC), including premedication and infusion-rate control when appropriate. Equally important, the systematic use of molecular, imaging, and immunologic biomarkers to stratify patients (e.g., by vascular permeability, immune phenotype, or anti-PEG/ABC status) will be critical to match specific nanotherapies to responsive subgroups, understand non-responders, and proactively manage safety. Within this context, batch-to-batch variability should be handled through clearly defined essential quality attributes (e.g., size/PDI, ζ-potential, drug loading/release, ligand density, endotoxin), orthogonal analytics, and a mechanism-linked potency assay, supported by formal comparability protocols during scale-up. Scalability should be demonstrated through reproducible unit operations (e.g., microfluidics, high-pressure homogenization, tangential-flow filtration, and lyophilization/reconstitution) within a defined design space, and with process analytical technologies to ensure product equivalence. Regulatory readiness can be strengthened by aligning development with QbD principles and ICH Q8/Q9/Q10/Q12, and by mapping our mechanism-to-metric framework onto PBPK to inform first-in-human dose selection and meet EMA/FDA expectations for nanomaterials. With these advances, and by embedding a concise “translational reality-check” within the framework, it can evolve from a descriptive synthesis into a practical decision aid for translational nanomedicine, supporting rational engineering to maximize therapeutic index while acknowledging uncertainty and biological heterogeneity, de-risking early clinical translation, and reducing trial-and-error in the development pipeline.

## Glossary

%ID/g: percent injected dose per gram
ABC: accelerated blood clearance
ADME: absorption, distribution, metabolism, and excretion
ApoA-I: apolipoprotein A-I
AUC: area under the plasma concentration–time curve
BBB: blood–brain barrier
BCS: Biopharmaceutics Classification System
CARPA: complement activation–related pseudoallergy
CL: clearance
COX-2: cyclooxygenase-2
**C**_**max**_: maximum plasma concentration
CYP3A4: cytochrome P450 3A4
DOX: doxorubicin
DTX: docetaxel
EPR: enhanced permeability and retention
F: oral (absolute) bioavailability
GI: gastrointestinal
IgG: immunoglobulin G
IL-6: interleukin-6
IV: intravenous
kPa: kilopascal(s)
MPS: mononuclear phagocyte system
NLC: nanostructured lipid carrier(s)
NP/NPs: nanoparticle(s)
NSCLC: non-small cell lung cancer
PBCA: poly(butyl cyanoacrylate)
PBPK: physiologically based pharmacokinetic (modeling)
PD: pharmacodynamics
PEG: polyethylene glycol
PEGylated: polyethylene-glycol–modified
PDI: polydispersity index
PK: pharmacokinetics
PLGA: poly(lactic-co-glycolic acid)
RES: reticuloendothelial system
SLN: solid lipid nanoparticle(s)
TAM(s): tumor-associated macrophage(s)
TMC–PA: N,N,N-trimethyl chitosan–palmitic acid (surface modification)
TNF-α: tumor necrosis factor-alpha
TPGS: D-α-tocopheryl polyethylene glycol 1,000 succinate
**t**_**½**_: terminal half-life
**V**_**d**_: volume of distribution
ζ-potential: zeta potential
